# Is There Anything to Smile about? A Review of Oral Care for Individuals with Intellectual and Developmental Disabilities

**DOI:** 10.1155/2012/860692

**Published:** 2012-02-28

**Authors:** Kathleen Fisher

**Affiliations:** College of Nursing and Health Professions, Drexel University, 245 N. 15th Street, Philadelphia, PA 19102, USA

## Abstract

Individuals with intellectual and developmental disabilities (I/DD) are at risk for dental disease and face substantial challenges in accessing both routine and preventive dental services. In terms of unmet needs it ranks third, following residential services and employment opportunities for this particular group of people. Poorer oral health status negatively impacts overall health and one's quality of life. Factors contributing to this problem include significantly higher rates of dental caries, periodontal disease, poor oral hygiene, low expectations, fear of treatment, and lack of awareness among individuals and carers. Additional factors include problems accessing dental care or denial of services because of inadequate education and clinical training, inappropriate bias, or inadequate levels of compensation to providers. Strategies to improve service delivery include individualized and coordinated care services, education of individuals, carers, and providers, including both classroom and clinical experiences with special needs patients in dental programs.

## 1. Introduction

 In 1961, President John F. Kennedy created this nation's first *President's Panel on Mental Retardation*. Like others with a family member with intellectual and developmental disabilities (I/DD) he was advocating for education, employment, community living, and engagement as well as research into the causes and prevention of I/DD. Ninety-five recommendations were put forth from this panel addressing issues from scientific research, civil rights, normalization, improved community services, and downsizing of institutional facilities [1]. Forty years later, Surgeon General David Satcher called on the experts including those with I/DD and their families, to identify and address the continued and multiple unmet health needs and disparities in health care experienced by individuals with I/DD. In their report titled *Closing the Gap: A National Blueprint to Improve the Health of Persons with Mental Retardation,* the following six goals highlighted the needs to (1) integrate health promotion into community environments of people with mental retardation, (2) increase knowledge and understanding for health and mental retardation, ensuring that knowledge is made practical and easy to use, (3) improve the quality of health care for people with mental retardation, (4) train health care providers in the care of adults and children with mental retardation, (5) ensure that health care financing produces good health outcomes for adults and children with mental retardation, and (6) increase sources of health care services for adults, adolescents, and children with mental retardation, ensuring that health care is easily accessible for them [2]. 

 Dramatic changes have occurred for individuals with I/DD since President Kennedy's call to action, including increases in life expectancy and shifts from living in institutions to living with family or in small community-based residences versus large state institutions [3, 4]. While some gains have been realized, individuals with I/DD continue to experience significant socioeconomic disadvantages and disparities in health care service delivery [1, 2, 4, 5]. Consequently, individuals with I/DD continue to experience significant unmet medical needs in general and significant unmet dental needs [6, 7] which is the focus of this paper.

## 2. Defining Intellectual and Developmental Disability

 Terminology has changed as well, and intellectual disability is now the preferred term to describe individuals with limitations in intellectual functioning and adaptive behavior [8, 9]. Hopefully this change can lessen the stigma attached to the previous term of *mental retardation*. Intellectual disability is considered a subset of persons who have developmental disabilities, defined as impaired cognitive functioning with onset during the developmental period from birth to age 22 [4]. Developmental disabilities cause functional limitations in three or more areas of life such as self-care, receptive and expressive language, learning, mobility, capacity for independent living, and economic self-sufficiency [4].

## 3. Prevalence

 According to the Centers for Disease Control, a total of 10 million Americans live with a developmental disability [10]. Analysis of data from the National Health Interview Surveys over 12 years (1997–2008), revealed an increase in the prevalence of any developmental disability from 12.84% to 15.04%, and a higher prevalence in boys compared to girls. For the years 2006 to 2008, one in six children reported a developmental disability. The prevalence for any developmental disability was 13.87% in this ongoing nationally representative sample. Attention Deficit Hyperactivity Disorder (6.69%) and autism (0.47%) showed significant and successive increases from 1997 to 2008, which researchers linked to the national campaigns to increase awareness of autism. Collinearity existed between learning disability (7.66%) and intellectual disability (0.71%); thus, learning disabilities were reported as a consequence of the intellectual disability versus a co-occurring condition. The researchers conclude that additional health, educational, and social support services would be needed to address these growing trends [11].

 A developmental disability requires a combination of interdisciplinary, generic care, treatment, and coordinated services to meet the individual's needs [12]. Some examples of developmental disabilities include autism, cerebral palsy, Down syndrome, epilepsy, blindness, deafness, and intellectual disability, and each can present with varying symptoms and severity. In addition developmental disabilities are characterized by significant limitations in three or more of the following areas: self-care, language, learning, mobility, and the capacity for independent living [12]. Intellectual disability, the focus of this paper, is identified by the age of 18 and characterized by limitation in intellectual functioning or cognition, that is, IQs of less than 70 which can influence one's learning ability, reasoning, and problem-solving skills [13].

 The number of people with I/DD is increasing because of population growth, better reporting, increased longevity, and the aging of this population, along with more accurate and sensitive methods of detection and diagnosis [14, 15]. These factors will result in an increased demand for all services including health, community (housing, employment, social supports), and dental care services [5, 7, 16].

 While individuals with I/DD deserve to have good oral health like anyone else, only pediatric dentistry includes individuals with special health care needs in its definition [5]. Dental care is one of the most common unmet health care needs for individuals with special needs [16]. Pediatric individuals with I/DD are often categorized as “special needs” with approximately 13% of Americans from birth to the age of 18 meeting the definition of a special needs child [17]. Reportedly, special needs children that begin care with a pediatric dentist never leave the practice, as dental care resources are limited for adults with I/DD [17].

 American dental schools offer approximately 580 postdoctoral programs in two categories: General Practice and Advanced Education in General Dentistry. These programs typically include 1-2 years of residency training [18]. Special Care Dentistry Programs are more recent in the field of dental practice and are intended to fill the gap in adult dental care for medically complex and special needs patients [16]. In 2006, the Commission on Dental Accreditation in the US adopted standards specifying that dental school graduates be competent in assessing treatment needs of patients with special needs [19]. Thus, all undergraduate programs must provide such education in their core curriculum. A debate as to the need for such a specialty is presented in “A Special Care Dentistry Specialty: Sounds Good, But…” [20]. These dentists argue that the need for services for those with I/DD will continue to increase and changes in educational requirements in dental schools should better prepare graduates for formal care. They suggest the reluctance to provide services among current providers may continue and is coupled with inadequate third-party coverage along with added chair time and provider effort. The authors portend that, along with other clinical dental specialties, the burden of providing the major component of care remains with the general practitioner [20].

## 4. Review

 The Global Oral Health Database and PUBMED were searched for the years 2001–2011 using the following keywords: intellectual disabilities, mental retardation, developmental disabilities, special needs, oral health, dental disease, prophylactic oral health care, and access to dental services. Forty papers were selected from the 524 abstracts reviewed, as they were in English, included research studies, clinical reviews, case studies, papers on best practice projects, and special needs curriculum in dental education. A series of six NIH continuing education booklets on oral care were reviewed which focused on autism, cerebral palsy, Down syndrome (DS), intellectual disability, developmental disability (DD), and physical disabilities. Federal guidelines including Healthy People 2020, and two Surgeon General Reports (Oral Health in America: A Report of the Surgeon General and Closing the Gap: A National Blueprint to Improve the Health of Persons with Mental Retardation) [2, 21–23], were also included in this paper.

## 5. Unmet Need for Dental Care and Services

 Individuals with intellectual and developmental disabilities (I/DD) are at risk for dental disease and face substantial challenges in accessing both routine and preventative dental services. In terms of unmet needs it ranks third, following residential services and employment opportunities for this particular group of people. The oral health status of individuals with I/DD remains a significant area of concern with negative impacts on one's overall health and one's quality of life [5, 16, 22].

 A myriad of issues contribute to poor oral health conditions and outcomes, although national studies to determine the actual prevalence of oral health disease among individuals with I/DD are lacking [20]. Periodontal disease, for example, is a serious and morbid oral condition among people with Down syndrome, with gingivitis and periodontitis beginning early and increasing in severity with age. These factors contribute to tooth loss among individuals with DS [24, 25]. According to the Surgeon General's Report on Oral Health in America, individuals with I/DD have significantly higher rates of dental caries, periodontal disease, and poor oral hygiene [22]. In addition, community-based residential facilities report inadequate access to dental care services as a significant issue. Data from the 2001 National Survey of Children with Special Health Care Needs was examined to explore the relationship between the receipt of routine medical and dental care among special needs children. An estimated 76% of parents reported the need for dental services in the prior 12 months, and of these 13% did not receive care. The researchers identified a link with lower income and failure to obtain routine medical care as a risk for failure to obtain dental care [26]. While access to dental care services presents its own challenges, individuals with I/DD vary considerably in their abilities to cooperate in the dental setting and treatment is usually more difficult when services are obtained. Physical or cognitive challenges may make performance and participation in oral hygiene difficult, including various behaviors like biting down on the toothbrush, refusing to open wide, or at all [16, 25, 27, 28]. It is also possible that their carers may have difficulty understanding or lack the physical ability to perform personal oral health preventive practices. In addition, some oral problems are exacerbated by associated medical problems or side effects of medication or can be caused by the disability itself [5, 6, 16].

Studies indicate that a number of individual/carer/professional and service barriers exist and contribute to oral health problems in this population. These can include an increased risk of dental disease, low expectations, fear of treatment, and a lack of awareness among individuals and carers [5, 7, 20, 29]. An additional barrier, reported by carers, was the social impact of the disability which means the carers were not sure how the individual would behave in public or at the dentist's office, and this presented an additional barrier to seeking dental care and services [16]. Other factors include problems accessing dental care or denial of services because of inadequate education and clinical training, inadequate levels of compensation to providers, and inappropriate bias. This bias in particular may suggest that oral health is a low priority for this particular group in the context of other social and medical challenges [7, 30].

## 6. Greater Risk and Greater Disease Burden

 Individuals with special needs have more dental disease, untreated caries, and missing teeth, and there is inadequate attention to preventing dental disease in this population [29]. Those with I/DD require greater attention to oral hygiene, and if this is neglected mucosal changes, dental disease, tooth loss, and difficulties in eating and speaking can follow [31]. Inherent risks for those with Down syndrome also include a tendency for mouth breathing which can impact saliva production, as decreased salivary secretion (Xerostomia) can contribute to oral mucosal changes, increased caries, difficulty swallowing, and chronic burning mouth syndrome [25, 31]. For individuals with cerebral palsy dental abrasion from gastroesophageal reflux is not uncommon [32]. In addition, individuals with I/DD are frequently prescribed medicine in syrup form, which can be high in sugar, exposing the teeth to increased risk of caries [5, 32]. According to reviews of dental practices, the pain, suffering, and stigma experienced by special needs clients is beyond that found in other segments of society and is often the result of ignorance, fear, stigma, misconceptions, and negative attitudes [6, 33].

 Dental disease for individuals with special needs is not peculiar to the US. Studies show that dental caries are the most prevalent disease among individuals with I/DD worldwide, and “dental treatment is the greatest unattended health need of the disabled” [34]. In the UK over 200,000 adults have profound learning disabilities and/or complex medical conditions with poorer oral health, poorer health outcomes, and poorer access to services compared to the rest of their population [35]. In a study of 112 individuals with DS in Kuwait, higher plaque scores and gingival irritation were found on initial screening which decreased after 3 months of a supervised brushing program in their school [36]. Poor oral health including signs of gingivitis and untreated caries were found by dentists on screenings of 1,286 Special Olympics athletes in Nigeria [37]. Sociodemographic and clinical variables were compared in 225 individuals with I/DD in India. Researchers concluded that the major factors contributing to poor oral health were age, that is, the oldest age group had poorer oral health; having Down syndrome and carers with low educational levels [34]. Irish researchers were concerned in their longitudinal study of 753 individuals with I/DD over the age of 40, that 16.5% of those studied had no teeth or dentures, and those with severe disability were twice as likely to have their teeth removed [15].

 Here, in the US, a three-group comparison study of periodontal status at three sites in Atlanta, of a Down's syndrome group (*N* = 55), and a non-Down ID group (*N* = 74) showed more missing teeth, caries, bleeding on exam, and higher gingival plaque as compared to the control group of 88 subjects [24]. Significant oral health disparities were found when 3 groups of North Carolinians were compared including 946 adults with I/DD, 1,598 adults with disability, and 4,358 nondisabled adults. Individuals with developmental disabilities were more likely than those with no disability to have never had their teeth cleaned or not to have had their teeth cleaned in the past five years. The researchers point out that this is an especially striking finding given that the nondisabled population group did not score high on those measures [37]. Unfortunately, as these studies suggest, the risk and need for routine and preventive dental services is greater in the I/DD population, while a significant oral health disparity exists when compared to the general population. Clearly those with I/DD could benefit from specific preventive oral health programs and routine screening for dental disease. 

## 7. Access Issues: Lack of Education, Reimbursement, Stigma

 Numerous reports cite lack of access to dental care and services as a critical problem for those with special needs [6, 16, 26]. Since the mid-1970s approximately two-thirds of individuals previously living in institutions were moved into the community. Researchers point out that access to dental care services was exacerbated by this deinstitutionalization movement, as once available services became unavailable in the new community locations [16, 27]. Further complicating the issue was the lack of dental providers trained to address the special needs of this population coupled with reimbursement or limited third-party support for complex services [16, 20, 29].

 The American Dental Association [18, 38] identified that the issue of limited access for those with I/DD to oral health care begins in dental schools which are providing minimal didactic and clinical experience in caring for special needs individuals. This they report causes dentists to be hesitant to treat these patients and practitioners are not prepared to provide needed services. A study of 295 third- and fourth-year dental students from five US programs reported receiving 5 hours or less of instruction devoted to caring for individuals with I/DD (68%), with 51% reporting no clinical training in this area at all [18, 38]. Dental student comfort level in treating vulnerable populations was assessed in a sample of 690 graduates from 1992 to 2004. In general, students reported that prior experience in school with this group had a positive impact on their comfort level in treating individuals with special needs [39]. Clearly, education and clinical experience in dental schools of individuals with intellectual and developmental disability should be addressed, especially given the increased need of this vulnerable population.

 A survey of 22 US and Canadian dental schools, for example, revealed that separate courses on special needs was offered in only 64% of programs, while clinical experience varied; that is, only 37% of the programs had a designated clinical area where their students could learn about special needs patients. The study concludes that if oral health disparities and access issues are to be addressed for this particular group, future research needs to focus on developing best practices in educational efforts and dental programs [32]. A review of clinical services in the UK also supports the need for special programs and the need to develop a specialized workforce to serve the needs of the most vulnerable sectors of the community currently being provided by a relatively small number of socially committed dentists [27].

 Clearly people with I/DD have difficulty obtaining dental care, and even some believe that dental practices in the US actively discriminate against people with disabilities, likely because they have a disability that makes the health care professional uncomfortable [27, 32].

## 8. Oral Hygiene

 Oral hygiene is often neglected in individuals with I/DD and obtaining access to good dental care is difficult [4, 16, 32]. A 3-year longitudinal study assessed risk factors and the oral health of 189 Japanese individuals with DS living in a nursing home. Study findings suggest that the most important factor for caries and tooth extraction prevention related to oral care practices versus the level of disability. This study also pointed out limitations to good oral hygiene practices including grimacing and spasticity of oral musculature, and difficulty using fluoride toothpaste if the individuals could not be taught to properly rinse out after brushing with fluoride [40]. Gagging when using a toothbrush is also a possibility, making the achievement of good oral hygiene difficult [5].

 A study of the oral health of over 1,000 Special Olympians in the UK identified the vulnerability of the older participants to dental problems. Gum inflammation was a common finding, and this study pointed to difficulty in maintaining surveillance as individuals' age or their informal carers become less able or available to oversee oral hygiene [41]. The National Institute of Dental and Craniofacial Research has developed a series of informational guidelines on practical oral care to assist both the professional and carers for individuals with I/DD [21].

## 9. Compensation Issues for Services

 Those with disabilities, account for a disproportionately large share of health care expenditures in every age group. Individuals with I/DD are much more likely to depend on public programs like Medicare or Medicaid to pay for health care, with an estimated 75% of individuals with developmental disabilities relying on government funding for medical and dental services [16]. Approximately 80% of those without disability are covered by private health insurance; only 44% of those with severe disability have private insurance, with approximately 40% having government insurance only and 17% with no insurance at all [16]. Needless to say, having insurance is significantly associated with more physician contacts, which has been associated with dental visits for individuals with disabilities [26].

## 10. Strategies for Improved Oral Health

 Review articles like this can raise awareness to the need for better education of providers, carers, and individuals to the need for oral hygiene, routine, and preventative dental care [22]. In a qualitative study of 10 carers representing 4 individuals with I/DD in Australia, positive outcomes were noted when promoting specific oral health and environmental considerations in the dental arena were considered. These included providing choice when possible, time, teaching skills, and communicating directly with the individual [42]. Best practice criteria were reviewed in all 50 states to assess how the Healthy People 2020 objectives were being met or how the practices were responding to the Surgeon General's report on oral health. The special needs practice approach is still in development but includes preparing the dental workforce, making the financing system more responsive, organizing community resources to increase accessibility and empowering individuals' parents and caregivers, and promoting advocacy [6].

 Research points to poorer oral health, more treatment needs and suggest that individuals with I/DD would benefit from frequent oral health assessments. Parents and carers need to be educated on the need to supervise tooth brushing irrespective of age, especially given that oral hygiene was shown to decrease or become poorer with age [43]. Parents and carers indicated that they needed appropriate and timely oral health information early in their child's life, along with access to sympathetic dentists who were good communicators and were well informed about I/DD [20].

 While poor dental care may or may not affect mortality, it certainly affects morbidity and adds to the poor health burden of those already suffering from an array of health concerns. Better oral hygiene and dental care would lead to an improved quality of life for those with intellectual and developmental disabilities. Let us give them something to smile about!
